# 2162: The impact of *Clostridium difficile* infection on disease severity in patients with inflammatory bowel disease

**DOI:** 10.1017/cts.2017.249

**Published:** 2018-05-10

**Authors:** Alyce J. M. Anderson, Claudia Ramos-Rivers, Benjamin Click, Debbie Cheng, Ioannis Koutroubakis, Jana Al Hashash, Michael Dunn, Marc Schwartz, Jason Swoger, Arthur Barrie, Miguel Regueiro, David Binion

**Affiliations:** University of Pittsburgh, Pittsburgh, PA, USA

## Abstract

OBJECTIVES/SPECIFIC AIMS: Inflammatory bowel disease (IBD) patients are at an increased risk of *Clostridium difficile* infection (CDI) but the impact of CDI on disease severity is unclear. The aim of this study was to determine the effect of CDI on long-term disease outcome in a cohort of IBD patients. METHODS/STUDY POPULATION: We analyzed patients enrolled in a prospective IBD natural history registry. Patients who tested positive at least once formed the CDI positive group. We generated a 2:1 propensity matched control cohort based on risk factors of CDI in the year before infection. Healthcare utilization data (emergency department use, subsequent hospitalizations, telephone encounters), medications, labs, disease activity, and quality of life metrics were temporally organized. RESULTS/ANTICIPATED RESULTS: A total of 198 patients (66 CDI, 132 matched controls) were included [56.6% female; 60.1% Crohn’s disease (CD), 39.9% ulcerative colitis (UC)]. Groups were not significantly different in the year before infection in all metrics but in the year of infection, having CDI was significantly associated with more steroid and antibiotic exposure, elevated C-reactive protein or erythrocyte sedimentation rate, and low vitamin D (all *p*<0.01). Infection was associated with increased disease activity metrics (UC: *p*=0.036, CD: *p*=0.003), worse disease-related quality of life (*p*=0.003), and increased healthcare utilization (*p*<0.001). In the next year after infection those with prior CDI continued to have increased exposure to vancomycin or fidaxomicin (*p*<0.001) and all other antibiotics (*p*=0.01). They also continued to have more clinic visits (*p*=0.006), telephone encounters (*p*=0.001), and worse disease-related quality of life (*p*=0.03), but disease activity and biomarkers of severity were not significantly different between groups. DISCUSSION/SIGNIFICANCE OF IMPACT: CDI infection in IBD is significantly associated with various surrogate markers of disease severity, increased healthcare utilization and poor quality of life during the year of infection. CDI patients continue to experience poor quality of life after infection with increased clinic visits and antibiotic exposure while disease activity is no longer significantly increased. These findings suggest that CDI infection may have a lasting effect on healthcare utilization beyond the acute treatment period.

